# OrangeExpDB: an integrative gene expression database for *Citrus* spp.

**DOI:** 10.1186/s12864-024-10445-5

**Published:** 2024-05-27

**Authors:** Chang Liu, Tingting Li, Licao Cui, Nian Wang, Guiyan Huang, Ruimin Li

**Affiliations:** 1 College of Life Sciences, Gannan Normal University, Ganzhou, Jiangxi 341000 China; 2 College of Agriculture, Northwest A&F University, Yangling, Shaanxi 712100 China; 3 College of Bioscience and Engineering, Jiangxi Agricultural University, Nanchang, Jiangxi 330045 China; 4 Citrus Research and Education Center, Department of Microbiology and Cell Science, IFAS, University of Florida, Lake Alfred, FL USA

**Keywords:** *Citrus*, Expression, RNA-sequencing, Database, OrangeExpDB

## Abstract

**Background:**

Citrus is a major fruit crop, and RNA-sequencing (RNA-seq) data can be utilized to investigate its gene functions, heredity, evolution, development, and the detection of genes linked to essential traits or resistance to pathogens. However, it is challenging to use the public RNA-seq datasets for researchers without bioinformatics training, and expertise.

**Results:**

OrangeExpDB is a web-based database that integrates transcriptome data of various *Citrus* spp., including *C. limon* (L.) Burm., *C. maxima* (Burm.) Merr., *C. reticulata* Blanco, *C. sinensis* (L.) Osbeck, and *Poncirus trifoliata* (L.) Raf., downloaded from the NCBI SRA database. It features a blast tool for browsing and searching, enabling quick download of expression matrices for different transcriptome samples. Expression of genes of interest can be easily generated by searching gene IDs or sequence similarity. Expression data in text format can be downloaded and presented as a heatmap, with additional sample information provided at the bottom of the webpage.

**Conclusions:**

Researchers can utilize OrangeExpDB to facilitate functional genomic analysis and identify key candidate genes, leveraging publicly available citrus RNA-seq datasets. OrangeExpDB can be accessed at http://www.orangeexpdb.com/.

**Supplementary Information:**

The online version contains supplementary material available at 10.1186/s12864-024-10445-5.

## Background

Belonging to the Rutaceae family, the Aurantioideae subfamily encompasses a variety of species, including *Citrus sinensis* and its related genera [1]. Oranges and their products are greatly appreciated for their nutritional, economic, and cultural advantages. Orange juice is one of the most beloved drinks [2]. It is widely known that tangerine peels have healing properties for medical purpose [3]. Fossil records from the late Miocene epoch in Lincang city, Yunnan province of China, suggest that a progenitor of the *Citrus* spp. have evolved approximately 8 million years ago [4]. Citrus plants, including oranges, lemons, and mandarins, are grown in more than 140 countries [5, 6]. Citrus growers are highly concerned about the outbreaks of citrus diseases, including Huanglongbing (HLB), which is the most devastating citrus disease [7]. *P. trifoliata* is often used as rootstock for *C. sinensis* and has displayed a certain level of tolerance to HLB [8]. Unfortunately, *Candidatus* Liberibacter asiaticus, *Ca*. L. africanus, and *Ca*. L. americanus, which are the causal agent of HLB, are yet to be cultured and there are no HLB-resistant citrus varieties [9]. The inability to culture the HLB pathogens renders it difficult to investigate the pathogenesis [10]. In addition, various issues need addressing, including yield, flavor, ripening time, stress resistance or tolerance, and mutation [11]. With the rapid progress of Next Generation Sequencing (NGS) technology, a vast quantity of Citrus transcriptome data has been collected, offering researchers the opportunity to address many challenging questions.

In the past decade, the advancement of NGS has generated a large amount of sequence data [12]. By 2023, more than 2,400 samples of *Citrus* spp. have been published in the NCBI Sequence Read Archive (SRA) database [13, 14]. In recent years, RNA-seq technology has been increasingly used to investigate various aspects of *Citrus* spp., such as gene expression changes caused by HLB [15–20]. Researchers have utilized this technique to build co-expression networks for analyzing core transcription factors in citrus development and stress responses [21, 22]. However, raw data for RNA-seq is scattered across multiple databases, including SRA, European Nucleotide Archive (ENA), and Genome Sequence Archive (GSA), making access difficult [23]. Furthermore, the data is often fragmented. As data growth continue to accelerate, there is a need for a standardized and simplified method of accessing gene expression data [24].

Recently, numerous online databases have been established, including CPBD [25], NGDC [26], TeaPGDB [27], BarleyExpDB [24], PlantcircBase [28], GRooT [29], PHI-base [30], and MPDB [31]. Notebly, these databases not only store data, but also offer various tools for analyzing biological data, such as BLAST [24, 25]. TeaPGDB, for example, is a user-friendly platform for tea plant genome, providing access to seven tea genome sequences and five tool sets, including “Gene Search”, “BLAST”, “JBrowse”, “SSR” and “Download” [27]. PlantcircBase is a database that consolidates all plant-circRNA data [28]. Importantly, BarleyExpDB contains transcriptional profiles of barley across various growth and developmental stages, tissues, and stress conditions [24]. These databases are useful for analyzing the intricate regulatory mechanisms of various organisms. Despite the availability of multiple genomes of available. sinensis, a comprehensive and centralized database of RNA-seq datasets for *Citrus* spp. is still lacking.

In this study, we have created OrangeExpDB, a database containing transcriptome data from 1,638 samples of five citrus species including *C. limon*, *C. maxima*, *C. reticulata*, *C. sinensis*, and *P. trifoliata* (Table 1). The expression profiles of genes in various tissues, developmental stages, and stress conditions can be easily downloaded and utilized. OrangeExpDB empowers researchers to access citrus RNA-seq data, thus facilitating the subsequent study of critical questions related to citrus.

**Table 1:** Statistics of BioProjects and BioSamples of *Citrus* spp. in this study

Species	BioProjects	BioSamples
*Citrus sinensis* (L.) Osbeck	61	920
*Citrus limon* (L.) Burm.	11	117
*Citrus maxima* (Burm.) Merr.	28	294
*Citrus reticulata* Blanco	19	182
*Poncirus trifoliata* (L.) Raf.	15	125

## Construction and contents

### Species selected

Our database includes five species of Citrus: *C. sinensis*, *C. limon*, *C. maxima*, *C. reticulata*, and *P. trifoliata*. *C. maxima*, *C. sinensis*, *C. limon*, and *C. reticulata* are severely affected by HLB [32–35], whereas *P. trifoliata* is often used as a rootstock and shows tolerance to HLB [8]. Therefore, we chose the five representative citrus species to construct the database.

### Acquisition of reference genomes

The Citrus Pan-Genome to Breeding Database (CPBD; http://citrus.hzau.edu.cn/download.php) [25] provides reference genomes and annotation information for *C. maxima* (http://citrus.hzau.edu.cn/data/Genome_info/HWB.v1.0/HWB.v1.0.genome.fa), *C. reticulata* (http://citrus.hzau.edu.cn/data/Genome_info/JZ.v1.0/JZ.v1.0.genome.fa), and *C. sinensis* (http://citrus.hzau.edu.cn/data/Genome_info/SWO.v3.0/SWO.v3.0.genome.fa). The reference genome and annotation information of *P. trifoliata* was downloaded from the Phytozome database v13 (https://phytozome-next.jgi.doe.gov/info/Ptrifoliata_v1_3_1). Genome sequences and annotation information of *C. limon* are available at citrusgenomedb (https://www.citrusgenomedb.org/Analysis/1470607).

### BioProjects and BioSamples

OrangeExpDB was created by collecting RNA-seq data from five species, resulting in a total of 134 studies (Table 1). *C. sinensis* had the highest number of studies at 61, followed by *C. maxima* with 28, *C. reticulata* with 19, *P. trifoliata* with 15, and *C. limon* with 11. The datasets for each species were categorized into several groups based on stages/tissues, mutants, and stress treatments.

### Collection and option

**Fig. 1:**
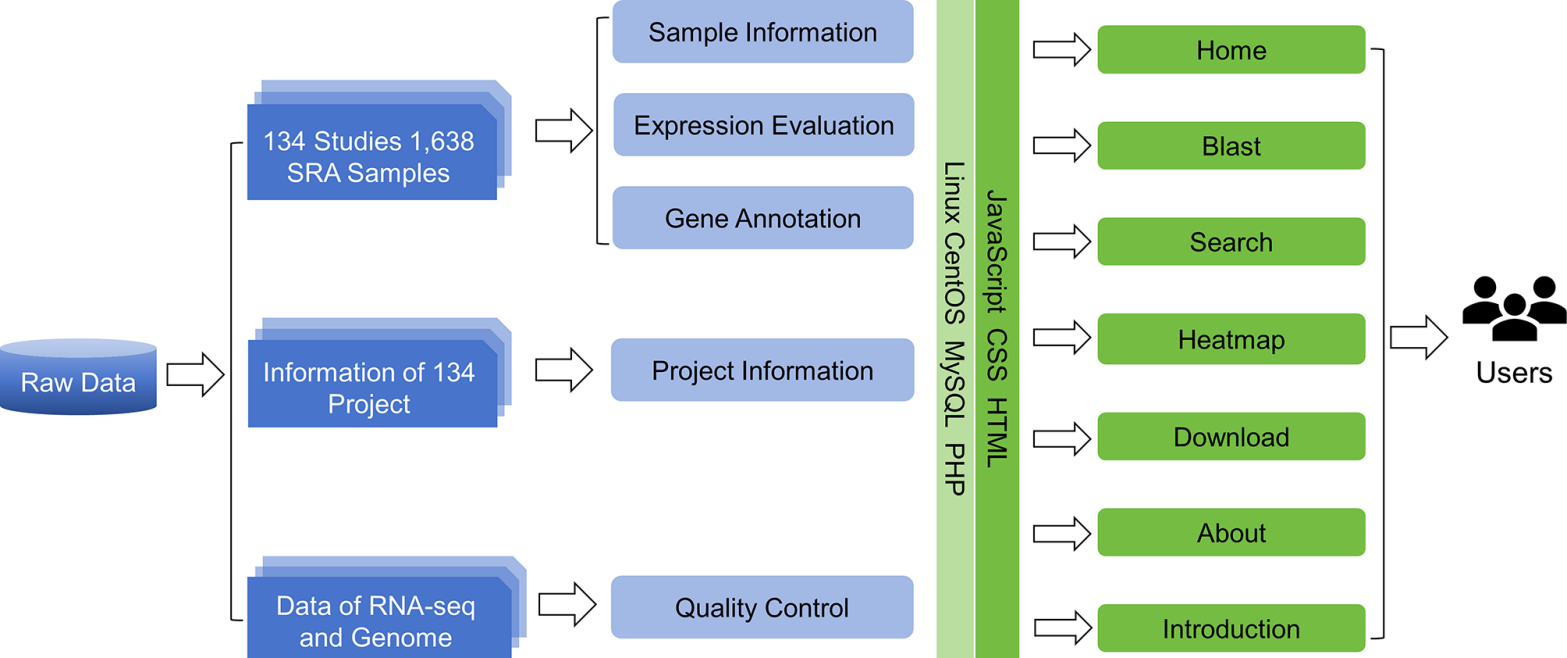
The procedure for creating the OrangeExpDB database. Raw sequencing data of RNA-seq studies were obtained and filtered, then aligned with the reference genome, resulting in a gene expression matrix. Gene functional information was subsequently annotated and functional modules such as “Blast”, “Search” and “Download” were added to the OrangeExpDB website

To streamline the screening process, we compiled information from NCBI and relevant literature, including project names, sample names, and library names. We also renamed some samples, of which the names were with unclear or ambiguous meanings, after checking relevant literature. Descriptions of each project were obtained. The RNA-seq raw data was downloaded from the NCBI SRA database and converted to fastq format using SRA toolkit v2.10.9 [36], resulting in a total of 1,638 samples (Table 1 and Supplementary Table 1). Adaptor and low-quantity sequences in the fastq files were removed using trimmomatic v0.39 [37]. HISAT2 was used to build the index for genomic assembly and comparison of RNA-seq reads. The resulting file was in SAM format, which was processed using samtools v1.13 with parameters ‘bS’ and ‘sort’ [38]. Finally, stringtie v2.2.1 and a self-written Python script (https://github.com/Viper-Chang/Batch-analysis-of-transcriptome-data) were used to extract the expression matrix of FPKM [39]. In order to provide convenience to users, we have also uploaded the TPM values of all genes for each species in our database at “DOWNLOAD” page. The heatmap on the webpage was created using plotly [40]. The matrix was stored, maintained and operated using MySQL v5.6.50 (Fig. 1).

## Database commons and interface

Our web server is hosted on Tencent Cloud’s lightweight application server, which is equipped with four Intel(R) Xeon(R) Platinum 8255 C CPUs clocked at 2.50 GHz and 8 GB of RAM. Access to the website is free of charge, as our purpose is not commercial. The Operating System (OS) running on the server is CentOS v7.9 (http://www.centos.org), a Linux-based OS. The web interface was designed using HTML (https://www.w3.org/html/), JavaScript (https://www.javascript.com/) and CSS (http://www.w3.org). The server-side back-end was encoded using PHP, and scripts were written in PHP to search data from MySQL and retrieve it to the front-end (Fig. 1).

## Community module

### Home

**Fig. 2:**
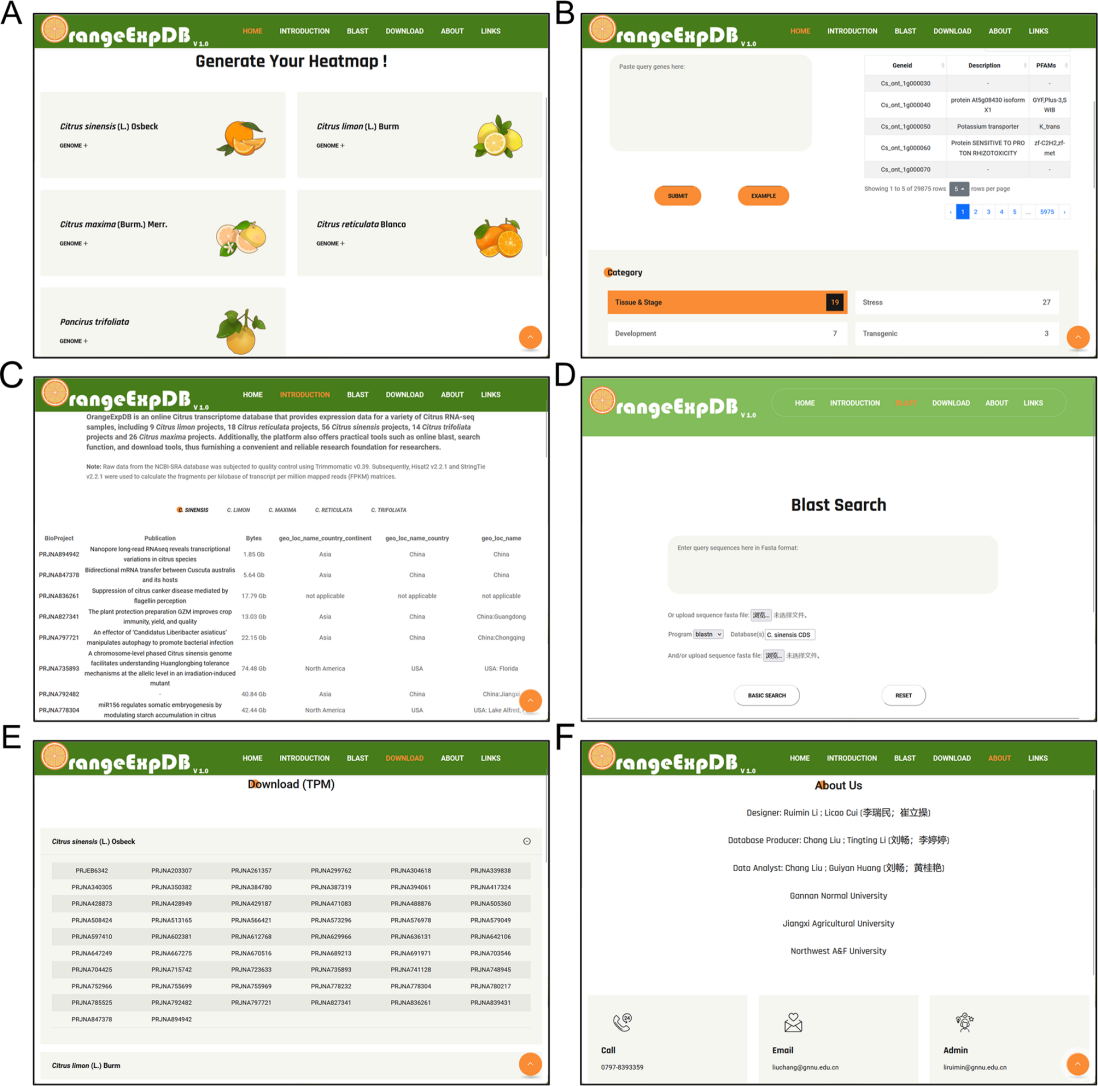
An overview of OrangeExpDB database. **A** and **B** Homepage of OrangeExpDB. **C** Introduction of OrangeExpDB. **D** Blast tools of OrangeExpDB. **E** Download page of OrangeExpDB. **F** Contact information and relevant hyperlinks

OrangeExpDB provides access to five citrus reference genomes including *C. sinensis* (reference genome: *Citrus sinensis* v3.0), *C. limon* (reference genome: *C. limon*_EMF-UC_v1-Primary_genome), *C. maxima* (reference genome: *Citrus grandis* (L.) Osbeck.cv.‘Cupi Majiayou’ v1.0), *C. reticulata* (reference genome: *Citrus reticulata* v1.0) and *P. trifoliata* (reference genome: *Poncirus trifoliata* v1.0). Each study is provided with a tag containing a summary of the study (Fig. 2A). A search box allows users to query gene expression of interest, accommodating up to 500 genes at a time. For more than 500 genes, users can submit multiple queries or download the raw data to extract the desired information (Fig. 2B).

### Introduction

OrangeExpDB provides a brief introduction and a drop-down menu for browsing the “Materials and Methods” used to construct the database. Users can access the analysis tools, t commands and parameters. Additionally, the interface provides a comprehensive description of each RNA-seq study, including sample accession number, stages/tissues, treatments, and other data (Fig. 2C).

### Blast tool

OrangeExpDB offers an online BLAST service for identifying genes with only sequence fragments and no gene IDs. Users can submit sequences in Fasta format, including amino acid and nucleotide sequences, or upload them in a text file format. Five BLAST algorithms (e.g., BLASTN, BLASTP, and TBLASTX) are available to identify possible homologous sequences. Results are displayed in order with the top candidates presented side-by-side for easy comparison (Fig. 2D).

### Downloads

On the “Download” page, users can download or re-analyze the matrix of FPKM and TPM values of interested BioProjects or BioSamples (Fig. 2E).

### About

The authors who contributed to the design and construction of the database are featured on the “About” page (Fig. 2F). Additionally, generic external links are accessible for further information.

### Links

The databases utilized in this study can be found on the “Links” page.

## An example for users

**Fig. 3:**
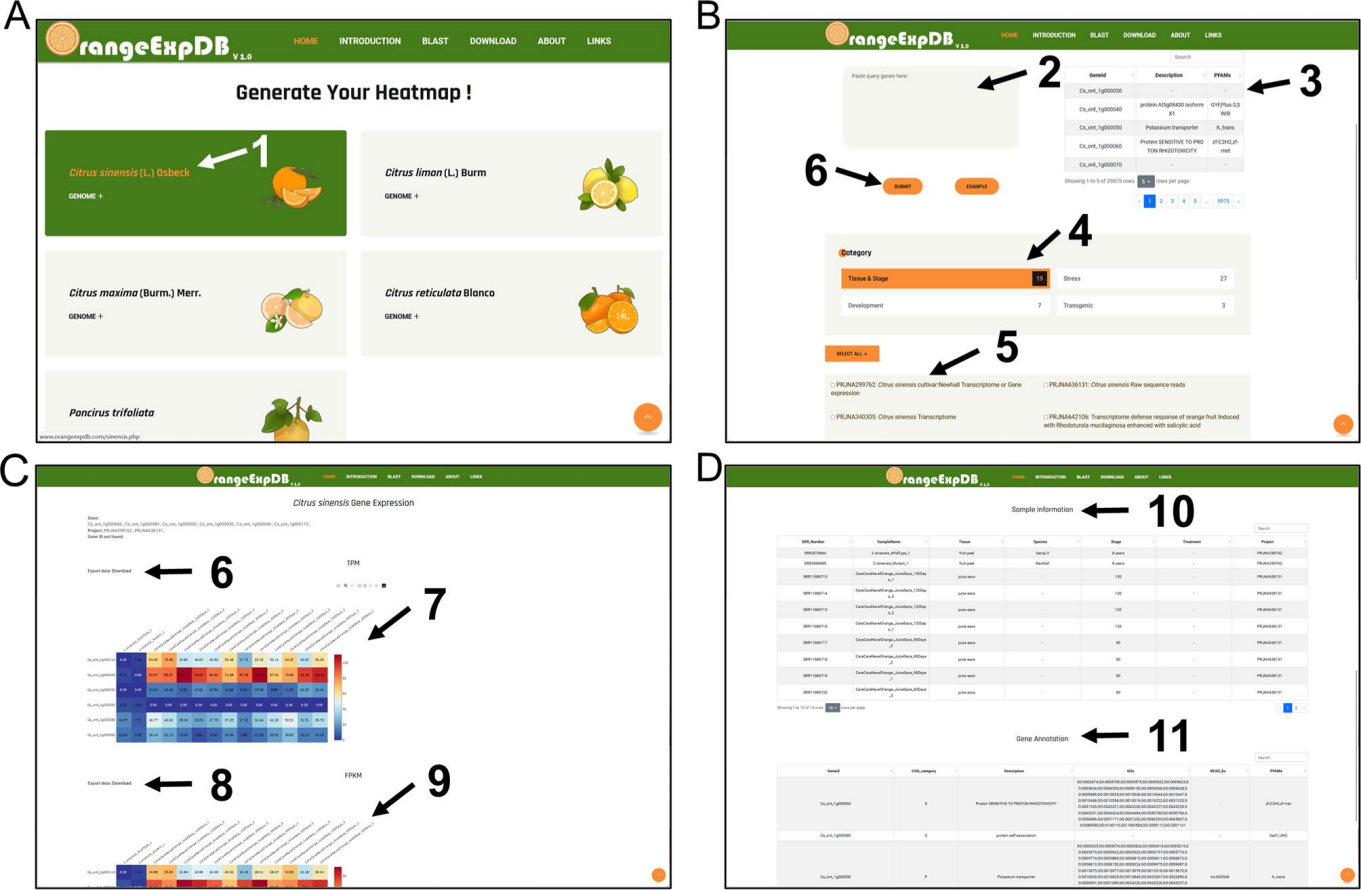
A demonstration of the usage of OrangeExpDB database. **A** Selection of interested species. **B** Each option for generating the expression value of candidate genes. **C** An example of the result page. **D** The sample information in the result page

To facilitate the use of the database, a straightforward example has been created to extract gene expression matrices of interest from selected BioSamples (Fig. 3). Users can select one of the five citrus Latin names displayed on the home page (Fig. 3A). Then, they can enter the identifiers of the desired gene locus, choose the category of the BioProjects, select the relevant BioSamples and submit (Fig. 3B). The results page displays detailed gene locus and BioProjects information, along with a download link containing the expression values of the specified genes from the selected BioSamples and a heatmap (Fig. 3C). Detailed information of the BioSamples including relevant publications, experiment accession, genotype/phenotype, stage/tissue and sequencing platform is presented at the bottom of the page (Fig. 3D).

## Prospects

OrangeExpDB is a dynamic database that offers convenient access to gene expression data for various citrus species. It will be regularly updated with the latest genomic information to ensure the accuracy of the expression matrix for each species. OrangeExpDB is designed to accommodate growing data and can be easily expanded. In future updates, the database will also include features for identifying RNA-editing sites and integrating single-cell RNA sequencing (scRNA-seq) data. Python scripts will be provided to simplify usage, and contributions from external groups and individuals are encouraged.

## Conclusions

OrangeExpDB is a comprehensive web-accessible database of RNA-seq data for citrus plants. It enables users to quickly search for information using known gene IDs, as well as providing expression levels of various tissues, developmental stages, and stresses. Additionally, the database provides useful tools such as function annotation, visualization, and result downloading. OrangeExpDB is a valuable resource for researchers looking to access and utilize transcriptome.

### Electronic supplementary material

Below is the link to the electronic supplementary material.

**Additional file 1:** Comprehensive description of the RNA-seq datasets obtained from the NCBI SRA database.

## Data Availability

The genomes of *C. sinensis*, *C. maxima*, and *C. reticulata* were obtained from CPBD (citrus.hzau.edu.cn/download.php), The genome of *P. trifoliata* was downloaded from Phytozome (https://phytozome-next.jgi.doe.gov/) and the *C. limon* genome was sourced from the link https://www.citrusgenomedb.org/citrus_downloads/Citrus_limon. The data for the study have been settled and, if a further investigation is required, readers can be referred to the corresponding authors. Publicly available RNA-seq datasets from the NCBI SRA were used to roughly estimate the expression pattern, with bio-project numbers PRJNA838230, PRJNA812325, PRJNA785525, PRJNA716747, PRJNA606613, PRJNA532796, PRJNA394067, PRJNA355134, PRJNA348468, PRJEB6342, PRJNA254441, PRJNA894942, PRJNA820365, PRJNA817805, PRJNA796621, PRJNA795605, PRJNA785525, PRJNA776249, PRJNA704217, PRJNA683589, PRJNA639316, PRJNA598932, PRJNA598773, PRJNA557834, PRJNA549576, PRJNA526584, PRJNA488908, PRJNA339650, PRJNA437176, PRJNA430310, PRJNA430306, PRJNA430145, PRJNA429973, PRJNA428873, PRJNA407231, PRJNA339838, PRJEB6342, PRJNA300206, PRJNA271737, PRJNA894942, PRJNA885437, PRJNA883800, PRJNA853264, PRJNA785525, PRJNA776912, PRJNA734968, PRJNA706142, PRJNA645612, PRJNA640485, PRJNA623065, PRJNA545864, PRJNA341756, PRJNA483477, PRJNA393067, PRJNA393070, PRJNA300206, PRJNA280255, PRJNA859629, PRJNA894942, PRJNA847378, PRJNA836261, PRJNA827341, PRJNA827342, PRJNA827343, PRJNA827344, PRJNA827345, PRJNA827346, PRJNA797721, PRJNA735893, PRJNA792482, PRJNA785525, PRJNA778304, PRJNA780217, PRJNA778232, PRJNA755969, PRJNA755699, PRJNA752966, PRJNA748945, PRJNA741128, PRJNA723633, PRJNA715742, PRJNA704425, PRJNA703546, PRJNA612768, PRJNA691971, PRJNA689213, PRJNA670516, PRJNA667275, PRJNA647249, PRJNA642106, PRJNA636131, PRJNA629966, PRJNA602381, PRJNA597410, PRJNA579049, PRJNA573296, PRJNA576978, PRJNA566421, PRJNA513165, PRJNA508424, PRJNA505360, PRJNA488876, PRJNA340305, PRJNA471083, PRJNA417324, PRJNA429187, PRJNA428949, PRJNA428873, PRJNA394061, PRJNA384780, PRJNA387319, PRJNA350382, PRJNA339838, PRJEB6342, PRJNA304618, PRJNA299762, PRJNA261357, PRJNA203307, PRJNA839431, PRJNA934070, PRJNA894942, PRJNA806490, PRJNA776912, PRJNA487128, PRJNA587875, PRJNA576788, PRJNA558461, PRJNA554373, PRJNA482734, PRJNA473568, PRJNA414000, PRJNA329194, PRJNA314020, PRJNA279929. The matrix of gene expression is composed of FPKM values from 134 studies, which are all accessible from OrangeExpDB.

## Abbreviations

BLAST: Basic Local Alignment Search Tool
FPKM: Fragments Per Kilobase of Transcript Per Million Mapped Reads
NCBI: National Center for Biotechnology Information
PCA: Principal Component Analysis
RNA-seq: RNA-sequencing
SRA: Sequence Read Archive
T2T: Telomere-to-Telomere
TPM: Transcripts per million

## References

[1] Oueslati A, Ollitrault F, Baraket G, Salhi-Hannachi A, Navarro L, Ollitrault P (2016). Towards a molecular taxonomic key of the Aurantioideae subfamily using chloroplastic SNP diagnostic markers of the main clades genotyped by competitive allele-specific PCR. BMC Genet.

[2] Farag MA, Abib B, Ayad L, Khattab AR (2020). Sweet and bitter oranges: an updated comparative review of their bioactives, nutrition, food quality, therapeutic merits and biowaste valorization practices. Food Chem.

[3] El Barnossi A, Moussaid F, Housseini AI (2021). Tangerine, banana and pomegranate peels valorisation for sustainable environment: a review. Biotechnol Rep.

[4] Wu GA, Terol J, Ibanez V, López-García A, Pérez-Román E, Borredá C, Domingo C, Tadeo FR, Carbonell-Caballero J, Alonso R (2018). Genomics of the origin and evolution of Citrus. Nature.

[5] Zhong G, Nicolosi E. Citrus origin, diffusion, and economic importance. citrus Genome 2020:5–21.

[6] Terol J, Soler G, Talon M, Cercos M (2010). The aconitate hydratase family from Citrus. BMC Plant Biol.

[7] Wang N, Trivedi P (2013). Citrus huanglongbing: a newly relevant disease presents unprecedented challenges. Phytopathology.

[8] Peng Z, Bredeson JV, Wu GA, Shu S, Rawat N, Du D, Parajuli S, Yu Q, You Q, Rokhsar DS (2020). A chromosome-scale reference genome of trifoliate orange (Poncirus trifoliata) provides insights into disease resistance, cold tolerance and genome evolution in Citrus. Plant J.

[9] Wang N (2019). The citrus huanglongbing crisis and potential solutions. Mol Plant.

[10] Zhou C (2020). The status of citrus huanglongbing in China. Trop Plant Pathol.

[11] Li Q, Qi J, Qin X, Dou W, Lei T, Hu A, Jia R, Jiang G, Zou X, Long Q. CitGVD: a comprehensive database of citrus genomic variations. Hortic Res 2020, 7. 10.1038/s41438-019-0234-3 PMC6994598 32025315

[12] Sahu PK, Sao R, Mondal S, Vishwakarma G, Gupta SK, Kumar V, Singh S, Sharma D, Das BK (2020). Next generation sequencing based forward genetic approaches for identification and mapping of causal mutations in crop plants: a comprehensive review. Plants.

[13] Katz K, Shutov O, Lapoint R, Kimelman M, Brister JR, O’Sullivan C (2022). The sequence read archive: a decade more of explosive growth. Nucleic Acids Res.

[14] Gao Y, Xu J, Li Z, Zhang Y, Riera N, Xiong Z, Ouyang Z, Liu X, Lu Z, Seymour D (2023). Citrus genomic resources unravel putative genetic determinants of Huanglongbing pathogenicity. iScience.

[15] Wang Y, Zhou L, Yu X, Stover E, Luo F, Duan Y (2016). Transcriptome profiling of Huanglongbing (HLB) tolerant and susceptible citrus plants reveals the role of basal resistance in HLB tolerance. Front Plant Sci.

[16] Balan B, Ibáñez AM, Dandekar AM, Caruso T, Martinelli F (2018). Identifying host molecular features strongly linked with responses to huanglongbing disease in citrus leaves. Front Plant Sci.

[17] Terol J, Tadeo F, Ventimilla D, Talon M (2016). An RNA-Seq‐based reference transcriptome for Citrus. Plant Biotechnol J.

[18] Deng B, Wang W, Deng L, Yao S, Ming J, Zeng K (2018). Comparative RNA-seq analysis of citrus fruit in response to infection with three major postharvest fungi. Postharvest Biol Technol.

[19] Ribeiro C, Xu J, Hendrich C, Pandey SS, Yu Q, Gmitter FG, Wang N (2023). Seasonal transcriptome profiling of susceptible and tolerant Citrus cultivars to Citrus Huanglongbing. Phytopathology.

[20] Pandey SS, Xu J, Achor DS, Li J, Wang N (2023). Microscopic and transcriptomic analyses of early events triggered by ‘Candidatus Liberibacter asiaticus’ in Young flushes of Huanglongbing-positive Citrus Trees. Phytopathology.

[21] Chen T, Niu J, Sun Z, Chen J, Wang Y, Chen J, Luan M (2023). Transcriptome Analysis and VIGS identification of key genes regulating citric acid metabolism in Citrus. Curr Issues Mol Biol.

[22] Rawat N, Kiran SP, Du D, Gmitter FG, Deng Z (2015). Comprehensive meta-analysis, co-expression, and miRNA nested network analysis identifies gene candidates in citrus against Huanglongbing disease. BMC Plant Biol.

[23] Deng CH, Naithani S, Kumari S, Cobo-Simón I, Quezada-Rodríguez EH, Skrabisova M, Gladman N, Correll MJ, Sikiru AB, Afuwape OO. Agricultural sciences in the big data era: Genotype and Phenotype Data Standardization, Utilization and Integration. 2023. 10.1093/database/baad088 PMC10712715 38079567

[24] Li T, Li Y, Shangguan H, Bian J, Luo R, Tian Y, Li Z, Nie X, Cui L (2023). BarleyExpDB: an integrative gene expression database for barley. BMC Plant Biol.

[25] Liu H, Wang X, Liu S, Huang Y, Guo Y-X, Xie W-Z, Liu H, ul Qamar MT, Xu Q, Chen L-L (2022). Citrus pan-genome to breeding database (CPBD): a comprehensive genome database for citrus breeding. Mol Plant.

[26] Members C-N (2023). Database resources of the National Genomics Data Center, China National Center for Bioinformation in 2023. Nucleic Acids Res.

[27] Lei X, Wang Y, Zhou Y, Chen Y, Chen H, Zou Z, Zhou L, Ma Y, Chen F, Fang W (2021). TeaPGDB: tea plant genome database. Beverage Plant Res.

[28] Xu X, Du T, Mao W, Li X, Ye C-Y, Zhu Q-H, Fan L, Chu Q. PlantcircBase 7.0: full-length transcripts and conservation of plant circRNAs. Plant Commun 2022, 3(4). 10.1016/j.xplc.2022.100343 PMC9284285 35637632

[29] Guerrero-Ramírez NR, Mommer L, Freschet GT, Iversen CM, McCormack ML, Kattge J, Poorter H, van Der Plas F, Bergmann J, Kuyper TW (2021). Global root traits (GRooT) database. Glob Ecol Biogeogr.

[30] Urban M, Cuzick A, Seager J, Wood V, Rutherford K, Venkatesh SY, De Silva N, Martinez MC, Pedro H, Yates AD (2020). PHI-base: the pathogen–host interactions database. Nucleic Acids Res.

[31] Hussain N, Chanda R, Abir RA, Mou MA, Hasan MK, Ashraf MA (2021). MPDB 2.0: a large scale and integrated medicinal plant database of Bangladesh. BMC Res Notes.

[32] Puttamuk T, Zhang S, Duan Y, Jantasorn A, Thaveechai N (2014). Effect of chemical treatments on ‘Candidatus Liberibacter asiaticus’ infected pomelo (Citrus maxima). Crop Prot.

[33] Hu J, Jiang J, Wang N (2018). Control of citrus Huanglongbing via trunk injection of plant defense activators and antibiotics. Phytopathology.

[34] Miles GP, Stover E, Ramadugu C, Keremane ML, Lee RF (2017). Apparent tolerance to huanglongbing in citrus and citrus-related germplasm. HortScience.

[35] Sajid A, Iftikhar Y, Ghazanfar MU, Mubeen M, Hussain Z, Moya-Elizondo EA (2022). Morpho-chemical characterization of Huanglongbing in mandarin (Citrus reticulata) and orange (Citrus sinensis) varieties from Pakistan. Chil J Agricultural Res.

[36] Leinonen R, Sugawara H, Shumway M (2011). The sequence read archive. Nucleic Acids Res.

[37] Bolger AM, Lohse M, Usadel B (2014). Trimmomatic: a flexible trimmer for Illumina sequence data. Bioinformatics.

[38] Li H, Handsaker B, Wysoker A, Fennell T, Ruan J, Homer N, Marth G, Abecasis G, Durbin R, Genome Project Data Processing S (2009). The sequence alignment/map format and SAMtools. Bioinformatics.

[39] Pertea M, Kim D, Pertea GM, Leek JT, Salzberg SL (2016). Transcript-level expression analysis of RNA-seq experiments with HISAT, StringTie and Ballgown. Nat Protoc.

[40] Sievert C. Interactive web-based data visualization with R, plotly, and shiny. CRC; 2020.

